# Gait Biomarkers Classification by Combining Assembled Algorithms and Deep Learning: Results of a Local Study

**DOI:** 10.1155/2019/3515268

**Published:** 2019-12-19

**Authors:** Eddy Sánchez-DelaCruz, Roberto Weber, R. R. Biswal, Jose Mejía, Gandhi Hernández-Chan, Heberto Gómez-Pozos

**Affiliations:** ^1^Departamento de Posgrado, Instituto Tecnológico Superior de Misantla, Veracruz, Mexico; ^2^Servicios Médicos, Universidad Juárez Autónoma de Tabasco, Villahermosa, Mexico; ^3^Tecnologico de Monterrey, Escuela de Ingeniería y Ciencias, Mexico; ^4^Universidad Autónoma de Ciudad Juárez, Ciudad Juárez, Mexico; ^5^Consejo Nacional de Ciencia y Tecnología, Centro de Investigación en Ciencias de la Información Geoespacial, Mexico City, Mexico; ^6^Universidad Autónoma del Estado de Hidalgo, Pachuca, Mexico

## Abstract

Machine learning, one of the core disciplines of artificial intelligence, is an approach whose main emphasis is analytical model building. In other words, machine learning enables an automaton to make its own decisions based on a previous training process. Machine learning has revolutionized every research sector, including health care, by providing precise and accurate decisions involving minimal human interventions through pattern recognition. This is emphasized in this research, which addresses the issue of “support for diabetic neuropathy (DN) recognition.” DN is a disease that affects a large proportion of the global population. In this research, we have used gait biomarkers of subjects representing a particular sector of population located in southern Mexico to identify persons suffering from DN. To do this, we used a home-made body sensor network to capture raw data of the walking pattern of individuals with and without DN. The information was then processed using three sampling criteria and 23 assembled classifiers, in combination with a deep learning algorithm. The architecture of the best combination was chosen and reconfigured for better performance. The results revealed a highly acceptable classification with greater than 85% accuracy when using these combined approaches.

## 1. Introduction

In Mexico, diabetes affects 60% of the population (http://fmdiabetes.org/wp-content/uploads/2014/11/diabetes2013INEGI.pdf). Diabetic neuropathy (DN) is a major consequence of diabetes mellitus and may have a detrimental effect on the patient's manner of walking, also known as “gait.” One variant of DN, diabetic peripheral neuropathy (DPN), is a peripheral pathology that causes the patient to show disorder in gait and progressive deterioration. Diagnosis of this pathology requires medical evaluation, but the use of computational techniques has also been proposed for its detection to reduce the margin of error of classification [1]. The present research involved the use of a network of sensors to acquire gait biomarkers for sample patients with DN and healthy individuals. These samples were used to create a model that contains the characteristics of healthy persons, as well as patients suffering from DN, and tags their state of health. Subsequently, a set of test data with the known health status of each case was used, but without tagging. The test data confirmed the efficiency of the models following the implementation of an exhaustive search that combined various algorithms (assembled classifiers + deep learning) and selection of the one with the maximum percentage of correctly classified instances. These instances showed with a high degree of certainty the existence of atrophy in muscles leading to an abnormal gait due to DN.

Machine learning has been widely used in several areas. In health research, it has been applied for disease diagnosis and the subsequent timely treatment of progressive diseases, including DN [2–5], which affects a high percentage of the world population. The present research focuses on the recognition of persons affected by DN through the classification of gait biomarkers. For this purpose, the following methodology was used: (i) A group of individuals with and without DN was selected. (ii) The sensors were placed, and the biomarkers data of gait were obtained. (iii) Each of the cases was tagged as positive or negative for DN, depending on whether the person presented the condition. (iv) The collected data were divided into two groups: the first was used as training data and the second one as test data. (v) A model that describes the behavior of the gait in both cases was built and trained with the training dataset. (vi) The model was evaluated using the test dataset (without tagging) and different classification algorithms (classifiers). (vii) The assembled classifiers were combined with a deep learning algorithm to find the one that generates the highest accuracy indexes.

In the state-of-the-art scientific literature, no method has yet combined these approaches to solve the problem presented here. In addition, due to the successive refinement obtained using this combined approach, the combination of an assembled classifier + deep learning algorithm appears to be a promising option for increasing the percentage of correctly classified instances by categorizing gait biomarkers in patients with DN against those of healthy controls.

## 2. State of the Art

DN is a consequence of degradation of the peripheral and autonomous nervous system. It is probably the most frequent complication of diabetes, affecting more than 50% of patients after 20 years of the disease course, depending on the severity and duration of hyperglycemia. The prevalence increases with years of progression, hyperglycemia, and established cardiovascular disease [6]. About 60 to 70 percent of people with diabetes suffer from some type of neuropathy, and these nerve disorders can develop at any time; however, the risk increases with age and with the duration of the disease. The highest DN incidence rates are found in people who have been suffering from diabetes for at least 25 years. DN also seems to be more common in people who have problems controlling their blood glucose (blood sugar), as well as in people with high levels of body fat or elevated blood pressure or who are overweight [7]. The DN is present in 40 to 50% of diabetic patients at 10 years after the onset of both type 1 and type 2 diabetes although less than 50% of these patients show DN symptoms. DN prevalence increases with the time of evolution of the disease and with the age of the patient, with its extent and severity related to the degree and duration of hyperglycemia [8].

There are several studies that propose the use of hardware devices to gather information from patients suffering from diseases that affect gait. In addition, a wide variety of machine learning algorithms have been used to categorize these diseases, some of which are described below.

Several studies have proposed the use of hardware devices to gather information about patients suffering from diseases that affect gait. In addition, a wide variety of machine learning algorithms have been used to categorize these diseases. For example, Mueller et al. compared gait characteristics, including torsional flexor pairs for feet and the range of ankle motion of subjects with diabetes mellitus and peripheral neuropathy. They found that patients with diabetes showed less mobility and lower ankle power, speed, and length of stride during walking, as well as a significant decrease in ankle strength and mobility, which seemed to be the key factors contributing to patterns of altered walk in these patients [9].

Similarly, Sacco and Amadio used sensitive time tracking in neuropathic and non-neuropathic diabetic patients as a measure of sensory deficit, focusing on dynamic and temporal parameters. The aim of their study was to investigate whether neuropathic patients develop changes in dynamics during walking to compensate for sensory deficits. They compared the results of neuropathic patients to those of a nondiabetic group to determine the relationships between the maximum plantar pressure cronaxie and sensitiveness in selected plantar areas, as they speculated that neuropathic patients develop compensatory musculoskeletal mechanisms to make up for their sensory deficit [10]. They based their research on an innovative thematic approach involving DPN and described and interpreted a treadmill self-healing system by neuropathic diabetic subjects using biomechanics and somatosensory considerations. Their innovation was the use of electromyography (EMG) and a treadmill, instrumented in a clinical application, to study and interpret motor control during gait in neuropathic diabetic patients. They found significantly higher somatosensory responses and pain tolerance thresholds in the diabetic neuropathic group; these responses were considered far from normal patterns. The EMG responses of the thigh and leg muscles, and especially the tibialis anterior and vastus lateralis, were delayed in the diabetic neuropathic group when compared to the normal pattern. The study showed that long-term sensory and motor defects altered muscle activation patterns during neuropathic walking on the treadmill [11].

Kwon et al. compared muscle activity and joint moments in the lower extremities when walking among nine subjects with DN and nine control subjects. They found that contraction of agonist and antagonist muscles occurred in the ankle and knee joints in subjects with DN during the support phase, and they concluded that these contractions may be related to an adaptive gait strategy that compensates for the decrease in sensory information from the ankle and foot. The contractions may contribute to a more stable gait, but the increased muscle activity probably has a higher energy cost. The differences in joint moments and electromyographic activity moment when walking in subjects with DN could be explained by several factors, including the loss of sensory perception, decreased muscle strength, decreased ankle mobility, and slow speed. The results also showed that subjects with DN had less ankle mobility, slower walking speeds, longer posture phases, and greater dorsiflexion of the lower peak ankle, ankle plantar flexion, and extension moments of knee when compared with the control subjects [12].

Yavuser et al. defined gait deviation in patients with diabetes mellitus by studying the associations between electrophysiological findings and gait characteristics. Their gait analysis showed a slow gait, shorter steps, limited knee and ankle mobility, lower plantar flexor moment of the ankle, and lower power in the diabetic group, and the differences were statistically significant. In addition, wave levels and latency were significantly correlated with ankle mobility and the plantar flexion moment of the ankle. They concluded that neuropathy might not be the only reason for gait deviations in patients with diabetes mellitus [13].

Akashi et al. compared the electromyographic activity of the thigh and calf muscles during gait in nondiabetic subjects and patients with DN at two stages of disease: those with and without previous experience of ulcers in their clinical history. They also investigated whether the changes in electromyography were due to some alteration in the reaction force on the floor during gaiting. They found that long-term neuropathic deficits, represented by a clinical history of at least one foot ulcer in the last two years, caused a late activation of the lateral vastus and lateral gastrocnemius and a lower propulsion of the vertical reaction force of the floor during barefoot walking [14].

Sawacha et al. investigated the muscle activity of deviations during gait, even in the early stages of diabetes, when neuropathy is absent. This study involved 50 subjects: 10 controls (body mass index 24.4 + 2.8, age 61.2 + 5.07), 20 diabetics (body mass index 26.4 + 2.5, age 56.53 + 13.29), and 20 neuropathic (body mass index 26.8 + 3.4, age 61.2 + 7.7). The electrical activity of six muscles was collected bilaterally in the lower extremity during the motion: gluteus medius, rectus femoris, tibialis anterior, long peroneus, gastrocnemius lateralis, and extensor digitorum communis, and the electromyographic activity was represented through a linear model. The time and space parameters were also evaluated by means of two Bertec force plates and a six-camera motion capture system (BTS, 60–120 Hz). In the initial contact and load response, an early response peak of rectus femoris activity occurred in diabetic subjects with and without neuropathy. The results suggest that important deviations of muscle activity are present in diabetic subjects although these are not directly related to neuropathy. The authors key finding can be considered as the presence of statistically significant alterations in non-neuropathic subjects. The results also suggest that important deviations of muscle activity are present in diabetic subjects although these are not directly related to the neuropathy. The authors believe that these results indicate that changes in the muscles of the foot occur before changes in nerve function can be detected. [15].

Deschamps et al. indicated that the reduction in the mobility of the foot was a key factor in the biomechanical alteration of the foot in individuals with diabetes mellitus. The aim of their study was to compare the kinematics and coupling in adult patients with diabetes, but with and without neuropathy, based on age, sex, and walking speed. Differences in the range of movement were quantified with the Rizzoli multisegment standing model, and different phases of the gait cycle were analyzed by repeated one-way measures using analysis of variance ANOVA. The groups with diabetes showed significantly lower values of movement compared to the control group. These findings suggested an alteration in the kinematics and segmental coupling during gait in diabetic patients with and without neuropathy [16].

Fernando et al. carried out a detailed review of electronic databases by searching for articles studying the effects of DN on gait. Their analysis of the spatial-temporal parameters, kinematics of lower limbs, kinetics, muscle activation, and plantar pressure showed that patients with DN had elevated plantar pressures and occupied a greater length of time in the stance phase with maximum contact in the flat feet position during gaiting, when compared to healthy controls [17].

Patterson and Caulfield used accelerometers to detect different gait conditions in people with normal and rigid ankles. They used an algorithm that quantifies the relevant characteristics of the swing phase in the foot and found a clear distinction between gait patterns in the ankle movements [18].

Gomes et al. studied patients with DN who suffered gait disturbances related to plantar ulcerations. They corroborated this relationship by designing computational simulations based on the gait muscle excitation patterns and found that their simulation was able to represent the hip posture adopted by patients with DN during movement as an adaptation to the loss of function in the distal muscles [19].

Sánchez-DelaCruz et al. proposed a classification model using gait information derived from data from a public repository for their tests and implementing various machine learning algorithms. The best result was obtained by combining the algorithms *LogitBoost* + *RandomSubSpace*, and they showed that assembled classifiers are a good alternative for binary classification [20]. Based on these results, they designed a sensor network for collecting gait biomarkers and built a database of patients with neurodegenerative diseases [21].

Camargo et al. designed a study to assess aspects of balance, ankle strength, and parameters of spatiotemporal gait in persons with DPN and to verify whether deficits in the parameters of the spatiotemporal gait were associated with muscular strength and ankle balance. Spatiotemporal mobility, functional mobility, balance performance, and ankle muscle strength were affected in individuals with DPN. The performance of the time up and go test and the isometric muscle strength of the ankle were associated with changes in spatiotemporal gait, especially during the condition of maximum gait velocity [22].

Berki and Davis collected pressure and tension data from 26 diabetic subjects and healthy controls using a new instrumentation that measures the vertical and horizontal force vectors of the plantar contact surface in the gait cycle. They applied two-dimensional discrete Fourier transform in each dataset, for each of the ten sensor sizes. The results showed that the sensor measuring 9.6 mm × 9.6 mm caused significant reductions in the three tension components (*p* < 0.001), while the sensors measuring 1.6 mm × 1.6 mm up to 4.8 mm × 4.8 mm can capture the entire spatial range of frequencies in the pressure and voltage data [23].

Anjaneya and Holi proposed a method that considers time and signal characteristics frequencies for DN classification using a neural network. Their approach was based on the fact that diabetes risks have increased among children and adults in the last decade, and that existing methods for early detection showed potential classification opportunities with an accuracy of 97.05% [24].

Al-Angari et al. used measures of shape and entropy to introduce new characteristics for capturing the variations in plantar pressure in a study of patients with DPN, retinopathy, and nephropathy compared with a diabetic control group without complications. The change in the position of the peak pressure of the plant with each step for both feet was represented as a convex polygon, asymmetry index, area of the convex polygon, second wavelet moment, and entropy of the sample [25].

Kavakiotis et al. carried out a systematic review of electronic information records of scientific articles of the last five years through the following queries: “Machine Learning AND Diabetes,” “Data Mining AND Diabetes” and “Diabetes,” whose revision was made in the PubMed and the DBLP *Computer Science Bibliography* databases. As a result, they found that different algorithms have been implemented with different datasets of diabetes. In their work, they presented a comparison of the percentages obtained in these studies [1].

The current state-of-the-art information indicates the following:

- The gait biomarkers, acquired by cameras or sensors, are a reliable source for the collection of gait information in people suffering from gait atrophy
- A large variety of machine learning algorithms have been used separately to classify disorders of the human gait
- Reliable and competitive classification percentages have been obtained

Given these observations, the classification of gait biomarkers of subjects with DN is an area that is expected to expand in such a way that reliable and accurate percentages of classification will be obtained. In the present study, we assumed that a sensor network would be a promising option for collecting gait information to build a dataset on which to implement an appropriate combination of machine learning algorithms.

## 3. Materials and Methods

### 3.1. Instrument to Collect Data

**Figure 1:**
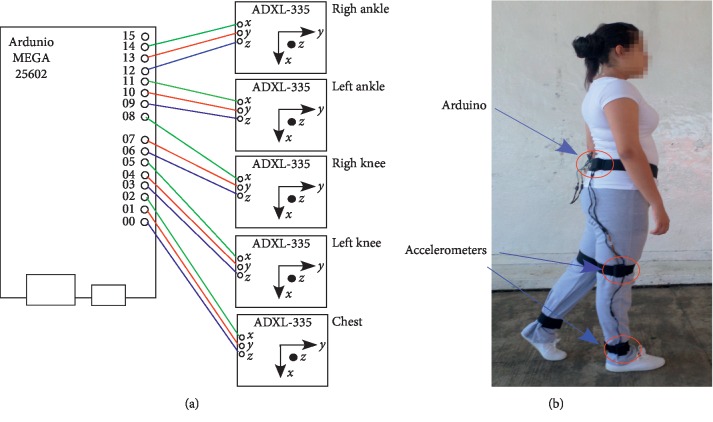
Sensor network: (a) topology; (b) validation.

A sensor network consisting of five 3-axis ADXL-335 accelerometer was built, validated, and connected to an Arduino MEGA-2560 card. The topological connections consisted of Cartesian coordinates *x*, *y*, and *z*, of the ground (GND) and a voltage of 3.3 V (Figure 1(a)). The sensors were distributed as follows: a sensor was placed on each ankle, on each knee, and on the hip (close to the gravity center). Data were acquired directly from the accelerometers, and no filter was used.

The ADXL-3351 accelerometer (http://www.analog.com/media/en/technical-documentation/data-sheets/ADXL335.pdf) is an analog sensor that detects movement; i.e., it is able to respond with an electrical signal to a disturbance induced by the application of a force or gravity. This device measures the acceleration on a 3G scale and uses a voltage level of 3.3 V. The Arduino MEGA-25602 (https://www.arduino.cc/en/Main/ArduinoBoardMega2560) is a card that contains, among others, 16 analog inputs, 4 UARTs (serial ports), a USB connection, a power connector, and a reset button. These electronic devices allowed the development of a useful and, above all, low-cost sensor network: 38.27 USD (Table 1).

A prototype of the sensor network was validated with a sociocultural gender group: boys and girls (Figure 1(b)). The data captured were clean; i.e., noise-free data were obtained, thus allowing an acceptable classification by combining the *LogitBoost* + *RandomForest* algorithms, as reported elsewhere [5].

### 3.2. Creation of the Database

The selection of subjects was based on the work presented in [26]. In that work, the authors referred to the creation of a dataset with human gait information and the effect of mechanical perturbations of fifteen subjects walking at three speeds on an instrumented treadmill.

Due to the characteristics of the subjects for our study, we opted to use the purposive sampling technique described in [27]. This is a nonprobability sampling that is highly effective when researchers need to study a certain domain as it allows them to use only those elements from the population that best suits the purpose of the study. This kind of sampling method is fundamental for the quality of data gathered because the reliability and competence of the source is controlled by the researchers, thereby providing an effective selection of the limited resources.

**Figure 2:**
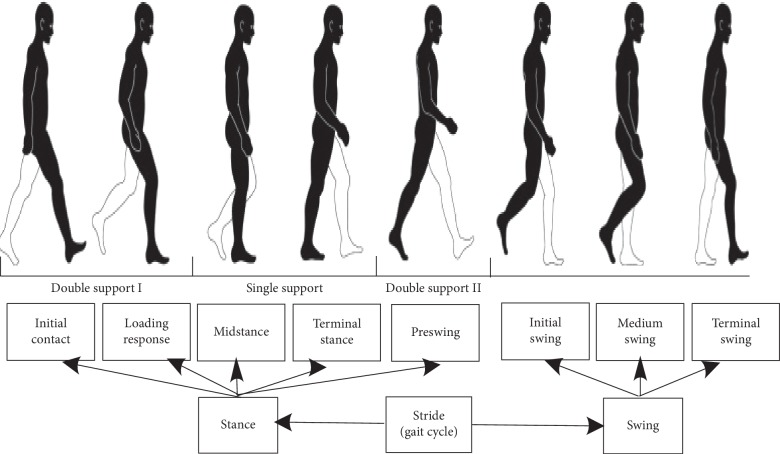
Gait cycle.

**Figure 3:**
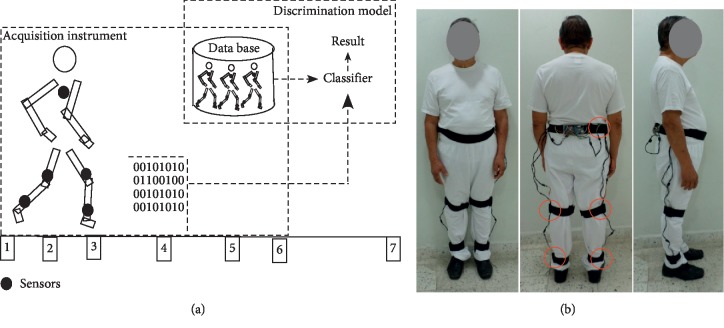
Gait laboratory [28]. (a) General classification model (gait lab); (b) capture of biomarkers.

In accordance with the gait cycle or stride, as shown in Figure 2, the database was created for patients suffering from DN using a sensor network. The data represented a particular region of the state of Tabasco, located in the southern zone of Mexico. For this purpose, a gait laboratory was created, consisting of a 20 m 3 m space with 8 m labelled for the track (Figure 3(a)) in the premises of the Medical Services Unit of the Autonomous University of Tabasco. The lab also had seating arrangements to allow the patients' caregivers to wait and to sign the consent report forms.

We worked with 10 patients who presented abnormality in gait due to DN, in addition to 5 healthy subjects (controls). The distribution of characteristics such as gender, age, weight, height, years of suffering, and cause is shown in Table 2. The inclusion criteria were any gender; age equal to or greater than 15 years; and ambulatory; i.e., they moved without support. We excluded patients who had experienced falls due to their condition, patients who did not sign *Informed Report*, pregnant women and patients with medical conditions that visibly did not allow them to walk for 5 minutes. Similar studies for gait analysis in patients have been published, for 13 subjects with amyotrophic lateral sclerosis [29], 14 subjects with Huntington's disease [30], 15 subjects related to Parkinson's disease [31], and 17 subjects with stroke [32].

The study subjects were instructed to walk normally to perform two familiarization trials with the sensor on prior to conducting the real test involving the capture of gait biomarkers (Figure 3(b)).

Therefore, one file was created for each patient with the raw data of the *x*, *y*, and *z* axes of each of the 5 accelerometers. These data were then used as inputs for the classifiers. In addition to each file, the attribute “case” was added, which refers to patients with DN pathologies or control subjects (Table 3). This resulted in the classes of binary sets: {diseased, control} with a total of 16 attributes.

### 3.3. Data Segmentation

**Figure 4:**
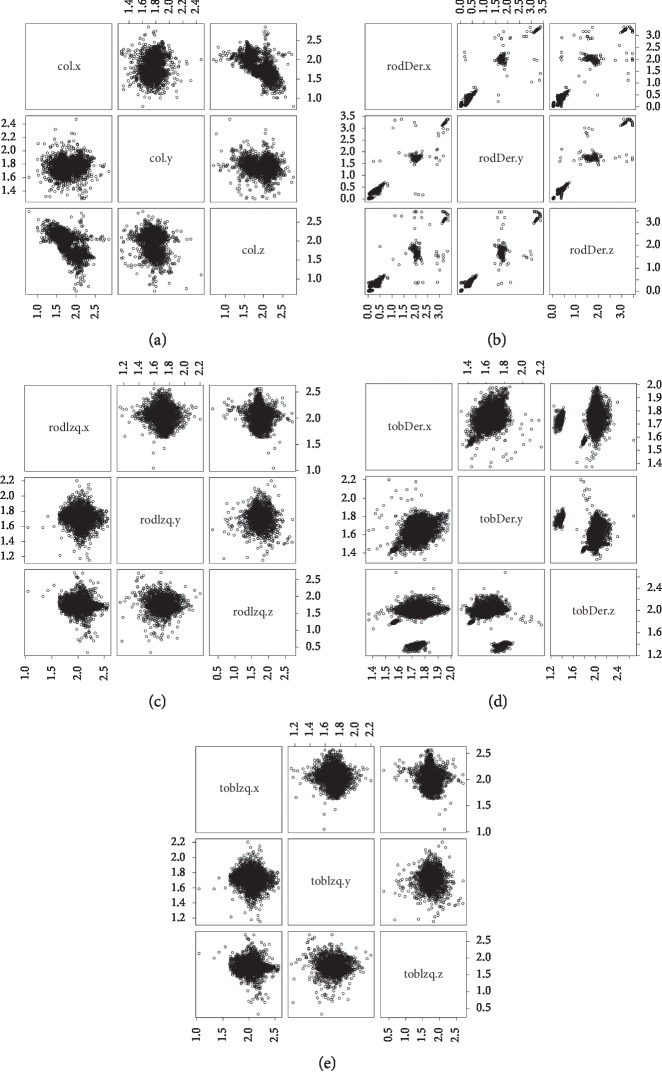
Correlation of Cartesian coordinates of each sensor: (a) gravity center; (b) right knee; (c) left knee; (d) right ankle; (e) left ankle.

For a visual quantitative analysis, the 10 files of the patients and the 5 files of the healthy controls were integrated into a single dataset, from which some statistical measurements (Table 4) and correlation (Figure 4) were obtained.

These measures *minimum*, *maximum*, *mean*, and *standard deviation*, facilitating correct data collection, i.e., the values oscillated in the same ranges, indicating no “outlier” noise. A relationship analysis of the attributes allowed the generation of correlation graphs of each sensor for all 15 study subjects (Figure 4).

Figure 4(a), which corresponds to the center of gravity, shows that no definite correlation exists between the Cartesian coordinates. Instead, the hip axes are grouped due to the linear displacement during gait. In relation to the knees, the right extremity (Figure 4(b)) shows a positive correlation and the left extremity (Figure 4(c)) depicts a grouping that corresponds to a weak relationship. In the right ankle (Figure 4(d)), a positive tendency is noted, while the left ankle (Figure 4(e)) denotes the presence of clustering. These observations confirm the assumption, derived from Table 4, that no addition or removal of attributes is required from the dataset.

### 3.4. Sampling Criteria

From the binary dataset, {diseased, control} was used to construct three subsets of data that considered the sampling criteria: cross-validation, 2/3–1/3, and representative sample.

- *Cross-validation*. The data were divided into *K* subsets (folds). One subset is used as test data and the rest (*K* − 1) as training data. The process was repeated during *K* iterations, with each of the possible test set. The error was calculated as the arithmetic mean of each iteration error to obtain a single result; therefore, if MSE_*i*_ (mean squared error) denotes the error in the *ith* iteration, then the cross-validation error is estimated by CV_(*k*)_=(i/*k*)∑_*i*=1_^*k*^MSE_*i*_.
- *2/3–1/3*. Another way to divide the data is in a training set *D*_train_ and its corresponding test set *D*_test_, such that *D*_train_ ∪ *D*_test_=*D* and *D*_train_∩*D*_test_=0. The model is trained in *D*_train_ to obtain $\hat{f}=D_{\text{train}}$ and calculate the generalization error using the data points in *D*_test_. The GE estimate (generalization error) is given by ${\hat{\text{GE}}}_{\text{hold}-\text{out}}=\hat{\text{GE}}\left(f_{D_{\text{train}}},D_{\text{test}}\right)$. This approach is also known as the hold-out method.
- *Representative Sample*. Statistical measure to obtain the test subset. This is obtained with the equation *n*=(*y*^2^pq*N*)/(*e*^2^(*N* − 1)+*y*^2^pq), where *n* = sample size, *y* = confidence level, *p* = probability of occurrence (0.50), *q* = probability of nonoccurrence (0.50), *N* = total population, and *e* = permissible error(0.1).

### 3.5. Classifiers

For each sampling subset (cross-validation, 2/3–1/3, and representative sample), 23 assembled algorithms were tested by combining them with the deep RNA *Multilayer perceptron*, known as the *Dl4jMlpClassifier* algorithm in *Waikato Environment for Knowledge Analysis* (WEKA):

1. AdaBoostM1 + Dl4jMlpClassifier,
2. AdditiveRegression + Dl4jMlpClassifier,
3. AttributeSelectedClassifier + Dl4jMlpClassifier,
4. Bagging + Dl4jMlpClassifier,
5. ClassificationViaClustering + Dl4jMlpClassifier,
6. ClassificationViaRegression + Dl4jMlpClassifier,
7. CostSensitiveClasifier + Dl4jMlpClassifier,
8. CVParameterelection + Dl4jMlpClassifier,
9. FilteredClassifier + Dl4jMlpClassifier,
10. LogitBoost + Dl4jMlpClassifier,
11. MetaCost + Dl4jMlpClassifier,
12. MultiClassClassifier + Dl4jMlpClassifier,
13. MultiClassClassifierUpdateable + Dl4jMlpClassifier,
14. MultiScheme + Dl4jMlpClassifier,
15. MultiSearch + Dl4jMlpClassifier,
16. OneClassClassifier + Dl4jMlpClassifier,
17. OrdinalClassClassifier + Dl4jMlpClassifier,
18. RandomCommittee + Dl4jMlpClassifier,
19. RandomizableFilteredClassifier + Dl4jMlpClassifier,
20. RandomSubSpace + Dl4jMlpClassifier,
21. Stacking + Dl4jMlpClassifier,
22. ThresholdSelector + Dl4jMlpClassifier,
23. WeightedInsta*ncesHandlerWrapper* + *Dl4jMlpClassifier.*

The combinations 2, 5, 7, 11, 13, 14, and 16 were discarded since the required nature of parameters could not be implemented. The tests with the other combinations revealed the best result with the representative sample test set and with the combination of *FilteredClassifier*+*Dl4jMlpClassifier* classifiers, which are described below.

- *FilteredClassifier*. This refers to a class in order to execute an arbitrary base classifier (in this case the *Dl4jMlpClassifier*) in data that have been passed through an arbitrary filter (in this case *Discretize* [33, 34], which discretizes a range of numeric attributes in the dataset in nominal attributes). Like the classifier, the filter structure is based exclusively on the training data, and the test instances are processed by the filter without changing its structure. If unequal instance weights or attribute weights are present and the filter or classifier cannot deal with them, the instances and/or attributes are resampled with replacement, based on the weights, before passing them to the filter or classifier (as appropriate).
- *Dl4jMlpClassifier*. This is based on the multilayer perceptron (Algorithm 1) and is an artificial neural network made of multiple layers. The neurons of the hidden layer use the weighted sum of the inputs with the synaptic weights *w*_*ij*_ as a rule of propagation, and on that weighted sum, a transfer function of sigmoid type or hyperbolic tangent is applied, which is bounded in response. The learning that is usually used in this type of networks is called backpropagation of the error. Both are increasing functions with two saturation levels: the maximum, which provides output 1, and the minimum, which provides output 0, for the sigmoidal function, and output −1, for the hyperbolic tangent.

### 3.6. Generation of Random Weights

A synaptic weight called {*w*_*i*,*j*_} is assigned for each input value. Although the values are assigned randomly, several methods exist in the literature to generate these values. One of them is Xavier's method [35], which was implemented in this study, as follows: Given a set of inputs {*x*_1_, *x*_2_,…, *x*_*n*_}, the weights of a distribution with zero mean and specific variance are initialized: Var(*W*)=(2/(*n*_in_+*n*_out_)), where Var(*W*) is the variance of the initialized weights with a normal distribution (usually Gaussian or uniform) for the neuron in question and *n*_in_ and *n*_out_ are the input and output number of neurons of a layer.

### 3.7. Base Function

The base function *f*=∑_*i*=1_^*n*^*w*_*i*_*x*_*i*_ is applied to the input values, with their assigned weights. In related work, the base function is also called the summation of initial values, the aggregation function, and the network function, among others, and, in general, can have different expressions.

### 3.8. Activation Function

Given the sum of initial values, the activation function is obtained, which is chosen according to the task to be performed by the neuron. For the multilayer perceptron, the most used activation functions are the sigmoidal function and the hyperbolic tangent function.

These functions have as image a continuous interval of values within the intervals [1,1] and [0,1], and they are given by the following equations: *f*_sigm(*x*)_=(1/(1+*e*^−*x*^)) and *f*_thip(*x*)_=((1 − *e*^−*x*^)/(1+*e*^−*x*^)). The activation function used in this research is discussed in Section 4.2.2.

### 3.9. Output Function

The output is given by the *Y*=*F*(*X*, *W*) function, where *Y* is the vector formed by the outputs of network (*y*_1_, *z*_2_, *y*_3_,…, *y*_*n*_), *X* is the input vector to network, *W* is the set of all the network parameters, i.e., weights and thresholds, and *F* is a nonlinear function.

### 3.10. Validation Metrics

To validate the results, the following techniques were used:

- Through the confusion matrix, each column represents the predictions of each class, while each row represents the instances in the real class. One of the benefits of the confusion matrix is that it allows to see if the model is confusing two classes, that is, recognizing one {class A} as other {class B}.
- Through the ROC space (receiver operating characteristic), which is elaborated from the *sensitivity* and *specificity* values.
- Validation of the medical specialist.

## 4. Results and Discussion

### 4.1. Combination of Assembled Algorithms and Deep Learning

The raw data from the dataset described in Section 3.2 were used for *Creation of the database*, and the binary tests were conducted {diseased, control}, as shown in Table 5. To do this, each assembled classifier of the WEKA family of metaclassifiers was combined with the deep learning algorithm, multilayer perceptron with backward propagation Dl4jMLPClassifier. The best result of the combination of *FilteredClassifier* + *Dl4jMlpClassifier* was obtained with the criterion of the representative sample.

The tests were performed using a Lenovo laptop G470, Intel (*R*) Celeron (*R*) CPU B800 @ 1.50 Hz, RAM 2.00 GB, 64 bit Operating System, Windows 7 Professional, with the WEKA (available from http://www.weka.org) tool developed by Witten and Frank [36].

### 4.2. Parameters Configuration of the Deep Learning Algorithm

#### 4.2.1. Iterations

**Figure 5:**
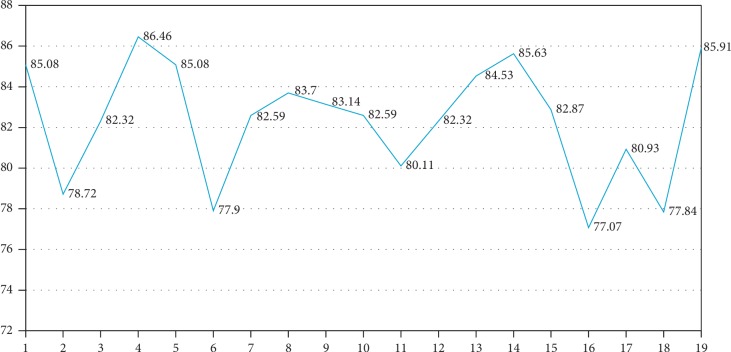
Iterations for training.

Table 5 shows that the best accuracy was 85.0829% with 10 iterations (*epochs*) for training, which is the preset configurational parameter in WEKA. The results were confirmed or improved by conducting the tests by increasing the iteration number to 20, 30, 40, 50, 60, 70, 80, 90, 100, 200, 300, 400, 500, 600, 700, 800, 900, and 1000 (Figure 5). Figure 5 does not show an elbow graph because the graph does not represent the search for the optimal number of elements for analysis; rather, it shows the maximum number of iterations of the algorithm needed to obtain the best performance.

The trend shows that, with 40 iterations, the percentage increases to 86.46% and does not show an increase in accuracy with higher iterations; thus, 40 iterations were considered as the ideal value.

#### 4.2.2. Activation Functions

The preset activation function in the WEKA tool is Softmax, which was used to obtain the maximum classification percentage, as mentioned before in section above. It was also tested with Cube, for 40 iterations and the percentage of classified instances decreased (see Table 6).

### 4.3. Validation Metrics

The results were validated using the following techniques.

#### 4.3.1. Confusion Matrix

Accuracy was calculated from the equation ((TP+TN)/total)(100), where TP are the true positives, TN are the true negatives, and total is the number of instances used for the test, that is, ((228+85)/362)(100)=86.46 (see values in Table 7).

Of the total number of test instances for the {diseased} class, 228 were classified correctly and 25 were confused with healthy controls. By contrast, 85 instances were correctly classified out of the control class and 24 were confused.

#### 4.3.2. ROC Space: Sensitivity and Specificity

The ROC space was elaborated considering the values of sensitivity and specificity, which were calculated from the confusion matrix, as follows: sensitivity=(TP/(TP+FN)) and specificity=1 − (FP/(FP+TN)), where TP were true positives, FN were false negatives, and FP were false positives. The above equations gave a specificity of 0.77 and a sensitivity of 0.90.

#### 4.3.3. Expert Opinion

The medical specialist (Dr. Roberto Germán Weber Burque Palacios), who validated this research based on his experience, notes that, at least for the study region, the precision of 86.46% is satisfactory for a first approach in this type of study concerning gait biomarkers in patients with DN. This corroborates the Swets affirmation: “In clinical diagnosis, when the *sensitivity* and specificity values represented in the Cartesian plane (or ROC space) exceed 0.8 to the left (*y* axis), it can be considered appropriate” [37].

In this research, patients and healthy individuals have been categorized with a high percentage of precision by applying a combination of assembled classifiers and deep learning to a dataset with gait biomarkers of DN. The expert suggested a future collection of more gait information of patients affected by DN, more healthy controls, and patients with another related disease that affects gait, to observe the performance of algorithm combination in a multiclass set.

Another recent study has shown a positive predictive value of 87% for detection of neuropathy in patients [38]. The classification is based on pseudomotor dysfunction; however, it requires a more expensive setup of equipment when compared with the cost of the sensors used here. One of the objectives of this study was to provide a low-cost tool for early identification of possible neuropathy. A limitation of the present study, which could be improved in future work, involves the details of the clinical characterization of the patients, such as the presence of diabetic complications. This information is important since complications can bias the results.

## 5. Conclusions and Future Work

The results presented here confirm the assumption that a combination of metaclassifiers with deep learning can generate a reliable and acceptable classification percentage of more than 85% by categorizing the gait biomarkers of affected subjects with DN and healthy controls. The best result obtained for the present study corresponds to the representative sample with 40 iterations. In addition, the convergence of disciplines is confirmed to help in solving complex problems—in this case, the categorization of DN.

The results were obtained from patients suffering from DN at different stages. Diagnosis of patients with DN at the early stages of disease is crucial, and the high sensitivity of the motion sensors can allow the detection of gait patterns that are otherwise imperceptible to the specialist.

The following seven efforts are considered worthwhile for the continuation and improvement of this research: (i) To corroborate the study with patients from other regions of Mexico, taking into consideration both DN cases and healthy controls, in order to build a dataset of greater dimensions and containing more information about gait biomarkers. (ii) To add sensors that record other parameters, such as heart rate, temperature, or others that provide additional relevant attributes and, if possible, that permit feature selection. (iii) To include information from other body limbs, such as the arms and neck. (iv) To develop an ad hoc expert system to support studies of diabetic diseases with atrophy factors in the patient's gait and/or to assist the specialist in predicting DN in persons, given the efficiency achieved by combining the metaclassifier with the deep learning algorithm *FilteredClassifier* + *Dl4jMlpClassifier*. This proposed expert system, motivated by the biometric recognition of Hernández et al. [39], could be used online with only basic and standard network protocols, without requirements for advanced network mechanisms (i.e., from the perspective of ubiquitous computing for a better experience for study subjects). (v) To improve the results by considering the implementation of the use of the method of *Combined selection and optimization of hyperparameters of classification algorithms* [40, 41], to explore the behavior of this method, and to increase the maximum percentage of 86.46% achieved in the present research. (vi) To extend this study to other ailments that cause immobility, such as osteoarthritis, as many other diseases are associated with movement disorders. (vii) To expand the database with more cases in future work.

## Figures and Tables

**Algorithm 1:**
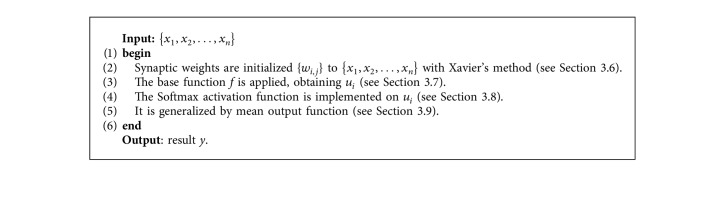
Multilayer perceptron.

**Table 1:** Cost of materials for the sensor network.

Device	Amounts	Price	Totals
Accelerometer ADXL-335	5	90.00	450.00
Arduino MEGA-2560	1	250.00	250.00
Wire-UTP cat. 5	10 m	2.00 e/m	20.00

Total in MXN			$ 720.00
Total in USD			$ 38.27

**Table 2:** Characteristic distribution of the study subjects.

Patient	Gender	Age	Weight (kg)	Height (cm)	Suffering years	Cause
1	M	54	89	1.70	5	Hereditary
2	M	60	108	1.65	10	Nutrition
3	F	56	99	1.60	4	Hereditary
4	M	56	81.5	1.62	6	Hereditary
5	M	62	73	1.57	15	Nutrition
6	F	50	70	1.59	8	Hereditary
7	M	58	102	1.61	6	Nutrition
8	M	57	87.7	1.58	8	Nutrition
9	F	61	90	1.65	3	Hereditary
10	M	50	83.2	1.63	5	Hereditary
11	F	35	72	1.61	0	Healthy
12	M	38	82	1.65	0	Healthy
13	M	45	95	1.67	0	Healthy
14	M	40	75	1.59	0	Healthy
15	F	29	59	1.55	0	Healthy

**Table 3:** Dataset attributes with gait biomarkers, fragment.

rodDer-X	rodDer-Y	rodDer-Z	rodIzq-X	rodIzq-Y	[…]	cad-Z	Case
1.87011821	2.08441092	2.06435782	2.3633454	…	…	…	Control
2.16604624	2.14561741	2.07332365	2.3833754	…	…	…	Control
2.06336545	2.13543299	2.07334487	2.3633567	…	…	…	Diseased
1.75786965	2.05353455	2.04234677	2.3733436	…	…	…	Diseased

**Table 4:** Statistical measurements of numerical attributes.

ValEstad	rodDer-X	rodDer-Y	rodDer-Z	rodIzq-X	rodIzq-Y	rodIzq-Z	tobDer-X	tobDer-Y	tobDer-Z	tobIzq-X	tobIzq-Y	tobIzq-Z	cad-X	cad-Y	cad-Z
Minimum	0.005	0.005	0.005	1.045	1.152	0.337	1.372	1.328	1.25	1.045	1.152	0.337	0.786	1.284	0.684
Maximum	3.359	3.379	3.472	2.568	2.202	2.705	1.982	2.202	2.681	2.568	2.202	2.705	2.852	2.471	2.778
Mean	0.464	0.448	0.446	2.029	1.711	1.702	1.745	1.638	1.952	2.029	1.711	1.712	1.779	1.762	1.95
EstDev	0.595	0.576	0.563	0.13	0.09	0.144	0.075	0.096	0.173	0.13	0.09	0.144	0.206	0.076	0.215

**Table 5:** Results of combining machine learning algorithms.

Metaclassifier	Deep learning	10-fold cross-validation	2/3–1/3	Representative sample
	Dl4jMlpClassifier	75.7	79.05	80.1105
AdaBoostM1	Dl4jMlpClassifier	76.6333	75.5	80.3867
AttributeSelectedClassifier	Dl4jMlpClassifier	77.3833	77.4	78.1768
Bagging	Dl4jMlpClassifier	79.4167	79.65	81.4917
ClassificationViaRegression	Dl4jMlpClassifier	66.6667	67.5	69.8895
CVParameterelection	Dl4jMlpClassifier	75.7	79.05	80.1105
*FilteredClassifier*	*Dl4jMlpClassifier*	*81.7333*	*84.5*	*85.0829*
LogitBoost	Dl4jMlpClassifier	66.6667	67.5	69.8895
MultiClassClassifier	Dl4jMlpClassifier	75.7	79.05	80.1105
MultiSearch	Dl4jMlpClassifier	75.7	79.05	80.1105
OrdinalClassClassifier	Dl4jMlpClassifier	75.7	79.05	80.1105
RandomCommittee	Dl4jMlpClassifier	79.1833	81.3	80.1105
RandomizableFilteredClassifier	Dl4jMlpClassifier	73.9	76.75	79.8343
RandomSubSpace	Dl4jMlpClassifier	79.7333	80.7	78.7293
Stacking	Dl4jMlpClassifier	36.6667	67.5	69.8895
ThresholdSelector	Dl4jMlpClassifier	72.9167	76.65	47.7901
WeightedInstancesHandlerWrapper	Dl4jMlpClassifier	75.7	79.05	80.1105

**Table 6:** Activation functions implemented and performance.

Activation functions	%
Softmax	86.46
Cube	79.27

**Table 7:** Confusion matrix for the binary set {diseased, control}.

Diseased	Healthy control	Classified as
228	25	Diseased
24	85	Healthy control

## Data Availability

The database used to support the findings of this study is available from the corresponding author upon request.
